# Wearing Garters

**Published:** 1874-06

**Authors:** 


### WEARING GARTERS.

It is almost peculiarly an American habit to wear garters below the knees. This constricts the great vein of the leg, interrupts the circulation, and tends to cause cold feet, which is the beginning of many troublesome ailments. In time the blood begins to dam up, forms ugly blue knots under the skin, known as varicose veins, which sometimes break and cause ulcerous sores for the remainder of life. If the garter is fastened above the knee all this is prevented.

Every prudent mother will impress this fact on the mind of her daughters.

